# Supplementary material for: Individual variation in Achilles tendon morphology and geometry changes susceptibility to injury
Source: eLife. 2021 Feb 16;10:e63204. doi: 10.7554/eLife.63204 (PMC7886322; doi:10.7554/eLife.63204)
Supplement: Supplementary file 1. — (File separated uploaded). [file elife-63204-supp1.docx]

Supplementary Table 1. Statistical analysis outcome of three sub-tendon mechanical properties.

|  | Kruskal-Wallis test | Pairwise Mann-Whitney U test | | | |
| --- | --- | --- | --- | --- | --- |
|  |  | LG – MG | LG – S | | MG – S |
| Cross-sectional area | | | | | |
| Test statistics | 10.820 | -1.776 | -2.611 | | -2.611 |
| DOF | 2.00 |  |  | |  |
| p-value | .004 | .095 | .008 | | .008 |
| Failure force |  |  |  | |  |
| Test statistics | 10.220 | -1.358 | -2.611 | | -2.611 |
| DOF | 2.00 |  |  | |  |
| p-value | .006 | .222 | .008 | | .008 |
| Ultimate stress | | | |  |  |
| Test statistics | .380 |  |  | |  |
| DOF | 2.00 |  |  | |  |
| p-value | .827 |  |  | |  |
| Ultimate strain | | | |  |  |
| Test statistics | 4.994 |  |  | |  |
| DOF | 2.00 |  |  | |  |
| p-value | .082 |  |  | |  |
| Young's modulus | | | |  |  |
| Test statistics | 1.040 |  |  | |  |
| DOF | 2.00 |  |  | |  |
| p-value | .595 |  |  | |  |
| Stiffness | | | |  |  |
| Test statistics | 10.220 | -1.358 | -2.611 | | -2.611 |
| DOF | 2.00 |  |  | |  |
| p-value | .006 | .222 | .008 | | .008 |
